# Spring Ailments

**Published:** 1877-05

**Authors:** 


### *SPRING AILMENTS.*

During the winter we require more food than in warm weather. It should consist mainly of those things that are rich in carbon, in order to supply the waste in the system, which is always proportionate to the low temperature to which our bodies are exposed. When the spring comes, and the temperature of the air undergoes a sudden change from cold to warm and "muggy" weather, the necessity and the desire for hearty food suddenly ceases, and the system, gorged with rich blood and deprived of the stimulus of cold weather to induce activity, is very much in the condition of a piece of "water-logged" timber. The vital forces are weaker than during any other season of the year; hence it is that the commencement of most of the diseases from which we suffer can be traced back to the early spring.

What we require is something which will arouse the dormant energies of the system, by gentle and rational means, bringing it gradually to a condition in conformity to the season and to what is required of it.

Goading ourselves or those under our control to over-exertion, before the system has had time to adapt itself to the changed condition of the season, is as cruel as it is irrational.

The surest and most pleasant way to escape the dangers of spring diseases is by a change of air, and the healthful exercise which a trip of a few days from the scenes of our labors and our cares involves.

Thoroughly convinced of the correctness of my theory in this respect, by repeated trials, I subjected it to another and successful test a few days ago.

Breathing, during most of the year, the salt air, and residing but a little above the sea level, the most complete change is to get the mountain air remote from the sea. For these reasons I prefer a trip by the Erie Railway; going always as far as Elmira, and, if I have the time to spare, as far as Buffalo; stopping on the way for a day or two at points of most interest. There is no other railroad from New York that so soon takes you from the dust and poisonous fumes of our always filthy streets. Even one week devoted to such a trip at any season, when we feel that we require a change, will do more than medicines to restore our flagging energies and our drooping spirits.

